# Supplementary material for: The role of node dynamics in shaping emergent functional connectivity patterns in the brain
Source: Netw Neurosci. 2020 May 1;4(2):467–83. doi: 10.1162/netn_a_00130 (PMC7286301; doi:10.1162/netn_a_00130)
Supplement: Supplementary file 1 [file netn-04-467-s001.pdf]

Forrester, M. & Crofts, J. J. (2020). Supporting information for "The role of node dynamics in shaping emergent functional connectivity patterns in the brain." *Network Neuroscience*, 4(2), 467–483. https://doi.org/10.1162/netn_a_00130

MATHEMATICAL METHODS

Network bifurcations of equilibria

Each node of the network is described by the m-dimensional Jansen–Rit model, with $m = 6$. There are $N = 78$ nodes in the network. Analysing bifurcations of network equilibria requires finding a set of $m \times N$ eigenvalues from the linearised system. Defining $\mathbf{y}_i \in \mathbb{R}^m$ as $(y_{1_i}, \ldots, y_{m_i})^\intercal$ allows us to write the system of first-order ODEs for the network as:

$$\frac{\mathrm{d}}{\mathrm{d}t}\mathbf{y}_i = M\mathbf{y}_i + B + Lf\left(\tau\mathbf{y}_i\right) + \varepsilon a A \sum_{j=1}^{N} w_{ij}\mathbf{K}(\mathbf{y}_j), \qquad i = 1, \ldots, N, \tag{1}$$

where

$$M = \begin{bmatrix} 0_3 & I_3 \\ M_{21} & M_{22} \end{bmatrix}, \qquad L = \begin{bmatrix} 0_3 & 0_3 \\ 0_3 & L_{22} \end{bmatrix}, \qquad \tau = \begin{bmatrix} 0_3 & 0_3 \\ \tau_{21} & 0_3 \end{bmatrix}, \tag{2}$$

and $B = (0, 0, 0, 0, AaP, 0)^\intercal$, $\mathbf{K}(\mathbf{y}) = (0, 0, 0, 0, f(y_1 - y_2), 0)^\intercal$, with

$$M_{21} = -\begin{bmatrix} a^2 & 0 & 0 \\ 0 & a^2 & 0 \\ 0 & 0 & b^2 \end{bmatrix}, \qquad M_{22} = -2\begin{bmatrix} a & 0 & 0 \\ 0 & a & 0 \\ 0 & 0 & b \end{bmatrix}, \tag{3}$$

$$L_{22} = \begin{bmatrix} Aa & 0 & 0 \\ 0 & AaC_2 & 0 \\ 0 & 0 & BbC_4 \end{bmatrix}, \qquad \tau_{21} = \begin{bmatrix} 0 & 1 & -1 \\ C_1 & 0 & 0 \\ C_3 & 0 & 0 \end{bmatrix}. \tag{4}$$

Here we have introduced the 3×3 identity matrix I_3, and the 3×3 zero matrix 0_3. The network steady state $\mathbf{y}_i = \overline{\mathbf{y}}_i$, for $i = 1, \ldots, N$, is defined by setting the left hand side of (1) to zero. We now linearise (1) by setting $\mathbf{y}_i(t) = \overline{\mathbf{y}}_i + \mathbf{u}_i(t)$, where $\mathbf{u}_i(t)$ is a small perturbation. This gives,

$$\frac{\mathrm{d}}{\mathrm{d}t}\mathbf{u}_i = \left[M + Lf'\left(\tau\overline{\mathbf{y}}_i\right)\tau\right]\mathbf{u}_i + \varepsilon a A \sum_{j=1}^{N} w_{ij} D\mathbf{K}(\overline{\mathbf{y}}_j)\mathbf{u}_j, \tag{5}$$

where $D\mathbf{K}(\mathbf{y}) \in \mathbb{R}^{m \times m}$ is the Jacobian of $\mathbf{K}(\mathbf{y})$. The only two non-zero entries of this matrix are given by $[\mathbf{K}(\mathbf{y})]_{5,2} = f'(\overline{y}_1 - \overline{y}_2) = -[\mathbf{K}(\mathbf{y})]_{5,3}$. It is now useful to define $D\mathbf{F}_i \equiv \left[M + Lf'\left(\tau\overline{\mathbf{y}}_i\right)\tau\right]$ and $D\mathbf{G}_j \equiv \varepsilon a A D\mathbf{K}(\overline{\mathbf{y}}_j)$, so that $D\mathbf{F}_i$ is the Jacobian which describes the intra-mass dynamics of node i and $D\mathbf{G}_j$ is the Jabobian for the effect of the inter-mass interactions with node j. Then we may write (5) in

the form

$$\frac{\mathrm{d}}{\mathrm{d}t}\mathbf{U} = \begin{bmatrix} D\mathbf{F}_1 & & 0 \\ & \ddots & \\ 0 & & D\mathbf{F}_N \end{bmatrix} \mathbf{U} + (w \otimes I_m) \begin{bmatrix} D\mathbf{G}_1 & & 0 \\ & \ddots & \\ 0 & & D\mathbf{G}_N \end{bmatrix} \mathbf{U}, \tag{6}$$

where $U = (\mathbf{u}_1, \ldots, \mathbf{u}_N)^\intercal$, and $\otimes$ denotes the tensor product. This system can be simplified by considering the eigenvalues of the connectivity matrix $w \in \mathbb{R}^{N \times N}$ (with components w_{ij}). We introduce a matrix of normalised eigenvectors, E, and a corresponding diagonal matrix of eigenvalues, $\Lambda = \mathrm{diag}(\mu_1 \ldots \mu_N)$, such that $wE = E\Lambda$. Imposing the change of variables $\mathbf{V} = (E \otimes I_m)^{-1}\mathbf{U}$ transforms (6) to

$$\begin{aligned} \frac{\mathrm{d}}{\mathrm{d}t}\mathbf{V} = (E \otimes I_m)^{-1} & \begin{bmatrix} D\mathbf{F}_1 & & 0 \\ & \ddots & \\ 0 & & D\mathbf{F}_N \end{bmatrix} (E \otimes I_m)\mathbf{V} \\ + (E \otimes I_m)^{-1}(w \otimes I_m) & \begin{bmatrix} D\mathbf{G}_1 & & 0 \\ & \ddots & \\ 0 & & D\mathbf{G}_N \end{bmatrix} (E \otimes I_m)\mathbf{V}. \end{aligned} \tag{7}$$

Assuming a homogeneous system such that $\bar{x}_i$ is independent of i, which is natural for identical units with a network connectivity with a row-sum constraint $\sum_{j=1}^{N} w_{ij} = \text{const}$ for all i, then we have a useful simplification $D\mathbf{F}_i = D\mathbf{F}$ and $D\mathbf{G}_i = D\mathbf{G}$ for all i. It is simple to establish that for any block diagonal matrix A, formed from N equal matrices of size $m \times m$, that $(E \otimes I_m)^{-1}A(E \otimes I_m) = A$. Moreover, using standard properties of the tensor operator, $(E \otimes I_m)^{-1}(w \otimes I_m) = (E^{-1}w) \otimes I_m = (\Lambda E^{-1}) \otimes I_m = (\Lambda \otimes I_m)(E^{-1} \otimes I_m)$. Hence, (7) becomes

$$\frac{\mathrm{d}}{\mathrm{d}t}\mathbf{V} = \begin{bmatrix} D\mathbf{F} & & 0 \\ & \ddots & \\ 0 & & D\mathbf{F} \end{bmatrix} \mathbf{V} + \begin{bmatrix} \mu_1 D\mathbf{G} & & 0 \\ & \ddots & \\ 0 & & \mu_N D\mathbf{G} \end{bmatrix} \mathbf{V}. \tag{8}$$

The system (8) is in a block diagonal form and so it is equivalent to the set of decoupled equations given by

$$\frac{\mathrm{d}}{\mathrm{d}t}\xi_p = [D\mathbf{F} + \mu_p D\mathbf{G}]\,\xi_p, \qquad \xi_p \in \mathbb{C}^m, \qquad p = 1, \ldots, N. \tag{9}$$

This has solutions of the form $\xi_p = \mathbf{A}_p \mathrm{e}^{\lambda t}$ for some amplitude vector $\mathbf{A}_p \in \mathbb{C}^m$. For a non-trivial set of solutions we require $\mathcal{E}(\lambda; p) = 0$ where

$$\mathcal{E}(\lambda; p) = \det\left[\lambda I_m - D\mathbf{F} - \mu_p D\mathbf{G}\right], \qquad p = 1, \ldots, N. \tag{10}$$

Solving $\mathcal{E} = 0$ for λ produces a set of eigenvalues which can be tracked to determine bifurcations. Since local stability requires the real part of all eigenvalues to be negative, if one of these eigenvalues crosses the imaginary axis the solution can undergo either a saddle-node bifurcation (Re $\lambda = 0 =$ Im (λ)) or a Hopf bifurcation (Re $\lambda = 0$, Im $(\lambda) \neq 0$).

Phase interaction function

To investigate the nature of phase-locked oscillatory states in the Jansen–Rit network, it is appropriate to use weakly-coupled oscillator theory. For a recent review see (Ashwin, Coombes, & Nicks, 2016). This gives rise to the set of equations where the phase interaction function H is determined in terms of two quantities. The first is the so-called phase response or adjoint $\mathbf{Q} \in \mathbb{R}^m$, that describes the response of an attracting limit cycle to a small perturbation. This can be computed by solving the *adjoint equation*. It is convenient to write the dynamics for a single uncoupled Jansen–Rit node in the form $\dot{\mathbf{y}} = \mathbf{F}(\mathbf{y})$, with $\mathbf{F}, \mathbf{y} \in \mathbb{R}^m$. Using the notation above we have explicitly that $\mathbf{F}(\mathbf{y}) = M\mathbf{y} + B + Lf(\tau\mathbf{y})$. The adjoint is given by the T-periodic solution of

$$\frac{\mathrm{d}}{\mathrm{d}t}\mathbf{Q} = -D\mathbf{F}^{\mathsf{T}}(\overline{\mathbf{y}}(t))\mathbf{Q}, \qquad \langle \mathbf{Q}(0), \mathbf{F}(\overline{\mathbf{y}}(0)) \rangle = \Omega. \tag{11}$$

Here $\overline{\mathbf{y}}(t)$ is a T-periodic of the Jansen–Rit node model and $\langle \; , \; \rangle$ denotes a Euclidean inner product between vectors. The second ingredient comes from writing the physical interactions in terms of phases rather than the original state variables. This is easily done by writing $\mathbf{y}_i(t) = \overline{\mathbf{y}}(\theta_i/\Omega)$. The phase interaction function is then obtained as

$$H(t) = \frac{1}{T}\int_0^T \mathrm{d}s \big\langle \mathbf{Q}(s), aA\mathbf{K}(\overline{\mathbf{y}}(s+t)) \big\rangle. \tag{12}$$

The adjoint equation is readily solved numerically by backward integration in time (Williams & Bowtell, 1997), whilst the integral in (12) can be evaluated using numerical quadrature.

Structural connectivity data

As described in ***Structural and functional connectivity***, we process structural connectivity data obtained from the HCP by thresholding, binarising and normalising by row. To confirm that these procedures do not unduly influence our conclusions, or restrict their applicability, we performed the following tests.

Statistical checks on the distribution of unthresholded SC weights indicate that node degree distributions have standard deviation of less than 10% of the mean, and outliers differ from the mean by less than 25% (data omitted). Therefore we are confident that our thresholding and binarisation process does not unduly influence the SC network structure, and thereby our results. As noted in the main text, we have also confirmed that the features of SC–FC correlation that we uncover in Fig. 5 are retained for different thresholds (namely: 20%, 30%, 40%; data not shown). To ensure that our modifications to the SC matrix did not crucially influence our findings, we recalculate equivalents of Figures 5(a) and (c) for a weighted, unnormalised network, obtaining similar SC–FC structures (see Figure 1). Inspection of node behaviour in the weighted un-normalised network, at parameter choices for which Figure 5(b) predicts stable or unstable synchronous behaviour, shows that the predictive power of our linear analysis is retained in the unnormalised case (data not shown).

As noted in Hansen, Battaglia, Spiegler, Deco, and Jirsa (2015), variation in coupling strength can affect SC–FC relations. In Fig. 2, we show that the essential organising features of the Jaccard similarity between SC and FC that we highlight in Fig. 5(a) of the main text are qualitatively unchanged for a range of choices of coupling strength ε.

REFERENCES

Ashwin, P., Coombes, S., & Nicks, R. (2016). Mathematical frameworks for oscillatory network dynamics in neuroscience. *The Journal of Mathematical Neuroscience*, *6*(1), 2.

Hansen, E. C., Battaglia, D., Spiegler, A., Deco, G., & Jirsa, V. K. (2015). Functional connectivity dynamics: modeling the switching behavior of the resting state. *NeuroImage*, *105*, 525–535.

Williams, T. L., & Bowtell, G. (1997). The calculation of frequency-shift functions for chains of coupled oscillators, with application to a network model of the lamprey locomotor pattern generator. *Journal of Computational Neuroscience*, *4*,

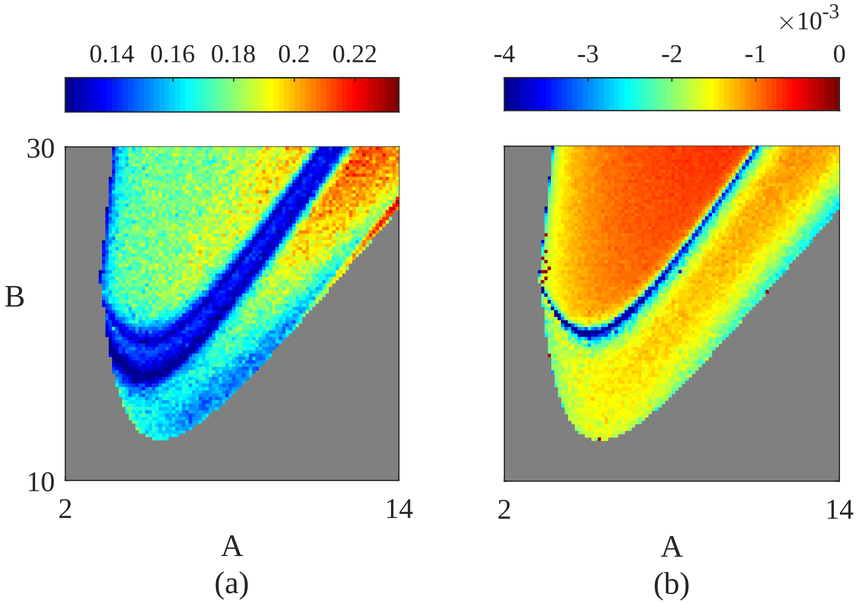

Figure 1. (a) Jaccard similarity coefficient between SC and FC in numerical simulations of the Jansen–Rit network model, when the network supports an oscillatory solution. Here the structural connectivity is the original weighted, un-normalised data. Model parameters are as in Fig. 5 (of main text). (b) The largest non-zero eigenvalue of the Jacobian for the full weakly-coupled oscillator network, calculated at a stable phase-locked state for the un-normalised SC matrix.

47-55.

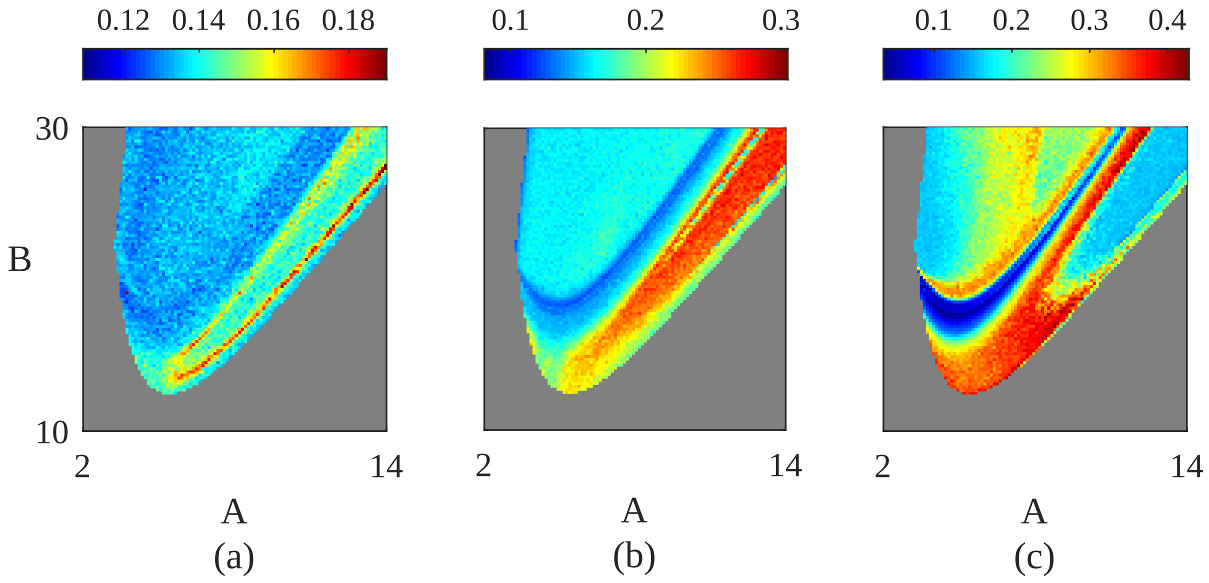

Figure 2. Jaccard similarity coefficient between SC and FC in numerical simulations of the Jansen–Rit network model, when the network supports an oscillatory solution. Parameters are as in Fig. 5 in the main text, except (a) $\varepsilon = 0.01$, (b) $\varepsilon = 0.1$, (c) $\varepsilon = 1.0$. Warmer colours indicate greater SC–FC correlation.
